# Failing professional practice placements in allied health: What do we understand about the student experience? A scoping review

**DOI:** 10.1007/s10459-023-10243-w

**Published:** 2023-06-07

**Authors:** Wendy Milgate, Jodie Copley, Jessica Hill

**Affiliations:** School of Health and Rehabilitation Sciences, Faculty of Health and Behavioural Sciences, University of Queensland, Brisbane, QLD Australia

**Keywords:** Allied health student, Failure, Fieldwork, Placement, Scoping review, Clinical placement

## Abstract

Professional practice placements are an essential component of allied health and nursing programs. Whilst most students pass these placements, a small percentage of students will fail or be at risk of failing. Supporting students undergoing a failing experience is a time critical, time consuming, emotional and resource-heavy task which is often undertaken by key university staff and impacts all stakeholders. Whilst several studies have provided insight into this experience from the educator and/or university perspective, this scoping review aimed to identify the students’ experience of failing or nearly failing a professional practice experience. Following Arskey and O’Malley’s framework for scoping reviews, 24 papers were included in this review. This review generated six themes including the reasons for failure, how failure looks and feels, how supports, service and strategies influence the student experience of failure, the importance of communication, relationships and organisational culture, the impact infrastructure and policies have, and the consequences of failure. The outcomes of this scoping review highlighted three key characteristics of the research to date: (a) the student voice is still largely missing; (b) the student perspective is distinctly different to that of other stakeholders; and (c) the interventions used appear not to be student-informed or student-led. Better understanding this experience from the student’s perspective could create a more sustainable practice education environment by designing and implementing more effective supports, services or strategies that reduce the overall impact a failing experience has on students and key stakeholders.

## Introduction

Professional practice experiences provide opportunities for students to integrate their theoretical knowledge into real-life work-based settings (Bissett et al., 2020; Isbel et al., 2020). Within the literature, professional practice has also been referred to as fieldwork, clinical placement, clinical education, practice education or work-integrated learning (Bissett et al., 2020; Cooper et al., 2010; Isbel et al., 2020; McGovern, 2021). Placements are an essential component of allied health and nursing programs to ensure that students develop their required professional competencies (McAllister & Nagarajan, 2015). The nature and format of these experiences varies across disciplines and programs but is typically conducted at an external agency with a qualified clinical educator who has disciplinary qualifications and experience in the relevant area of practice (McAllister & Nagarajan, 2015). University programs coordinate and support placements through dedicated staff who manage and support student placements including when a student is underperforming or failing (Lawton et al., 2021; Power & Albaradura, 2018; Stutz-Tanenbaum et al., 2015).

A student’s performance on placement is formally evaluated by the educator using the relevant university’s evaluation tool (McAllister & Nagarajan, 2015). Whilst most students pass their placement, the rates of failure in allied health and nursing programs have been reported to range from 1 to 32% (American Occupational Therapy Association, 2018; Attrill et al., 2012; Brandon & Davies, 1979; Foote, 2015; Gutman et al., 1998; Johnston et al., 2018).

Failing placement is a complex phenomenon and the impact of supporting an underperforming student has been identified as a time consuming and difficult experience for educators (Drake & Irurita, 1997; Hughes et al., 2016, 2021; Ilott, 1996; Larocque & Luhanga, 2013; McGovern, 2021; Nicola-Richmond et al., 2017; Power & Albaradura, 2018; Schaub & Dalrymple, 2013; So et al., 2019) and/or university staff (Hughes et al., 2021; Larocque & Luhanga, 2013; McGovern, 2021; Nicola-Richmond et al., 2017). Failing a placement can be due to various factors involving several stakeholders and has been demonstrated to have academic, financial, and emotional consequences for the student (Burgess, Phillips, et al., 1998; Davenport et al., 2018; Foo et al., 2017; Larocque & Luhanga, 2013). Whilst the impact for the student individually can be significant, the impact on other stakeholders is also important to consider. Supporting students undergoing a failing experience is a time critical, time consuming, emotional and resource-heavy task often undertaken by key university staff who already have high workload pressures (Stutz-Tanenbaum et al., 2015).

Whilst several studies have provided insight into this experience from the educator and/or the university perspective, the student voice from an allied health or nursing perspective on this matter appears to be less prominent in the literature (Davenport et al., 2018). For stakeholders to better support students through this experience, it may be important to apply a person-centred approach so that the student remains central to any decisions made, and their experiences, values and needs are considered in the design and delivery of supports offered to ensure the most effective outcomes (McCormack et al., 2017). Better understanding this experience in relation to what are the underlying variables that may contribute to success or failure, and to understand this experience from the student’s perspective, will inform all stakeholders on how to better support students who are undergoing a failure experience in a sustainable manner.

## Methods

Scoping reviews can be used to identify the gaps in the literature on a research topic, to identify the types of evidence that currently exist, the types of research conducted in this field, and whether a systematic review is warranted (Munn et al., 2018). Due to the apparent lack of literature that included the student’s voice, and that we wanted to consider a variety of study designs, we determined a scoping review to be the most appropriate methodology (Arksey & O’Malley, 2005; Munn et al., 2018). This study followed the six stage framework for conducting scoping reviews (Arksey & O’Malley, 2005; Levac et al., 2010): (1) identify the research question, (2) search and identify relevant studies, (3) chart the data, (4) collate, (5) summarise and (6) report the results.

The aim of this scoping review was to address the research question: “What is known about the experience of allied health and nursing students who fail or are at risk of failing a professional practice experience?”

To identify available evidence, five databases were systematically searched: CINAHL, MEDLINE, APA PsycNET (APA PsycInfo), EMBASE and Scopus. In consultation with the research librarian, the following original search terms were used across all fields to generate recommended mesh headings and preferred search terms – students, ‘allied health students’, fieldwork, placement, ‘clinical placement’, clinical education, and fail* or ‘at risk’. As definitions of allied health differ and typically include large numbers of disciplines (Allied Health Professionals Australia, 2023; Association of Allied Health Professionals, 2023) it was considered beyond the scope of the study to include each discipline as a separate term in the search. As some allied health disciplines were poorly represented in the initial search results, the disciplines of occupational therapy, physiotherapy, speech therapy and social work were added as search terms to yield more relevant results. The term clinical education was excluded due to the generation of a significant number of irrelevant results that related to graduate experiences.

The final search terms used were: (Students, Undergraduate OR Students, Health Occupations OR Students, Allied Health OR Students, Occupational Therapy OR Students, Physical Therapy OR Students, Speech-Language Pathology OR Students, Social Work OR Pre-entry) AND (student placement OR fieldwork OR professional practice OR work integrated learning) AND (fail* OR at risk). Minor changes to these search terms were applied in MEDLINE and APA PsycNET to yield relevant results. Results were limited to English language papers with no further limiters applied. The initial search was conducted in June 2021 and repeated in July 2022. The complete list of search terms as applied in CINAHL can be found in the Appendix.

Papers were included if they met the following criteria: (1) focused on undergraduate or graduate entry allied health or nursing students; (2) related to the student experience on placement; (3) related to students having challenges fulfilling the requirements of placement, failing the placement or being at risk of failing placement; and (4) reports or describes a research study. Papers were excluded if they focused on (1) services delivered by students; (2) evaluation of a tool used to assess student performance; (3) the process of students developing a specific clinical skill, knowledge base or competency; or (4) a model of support or preparation training for placement.

**Fig. 1:**
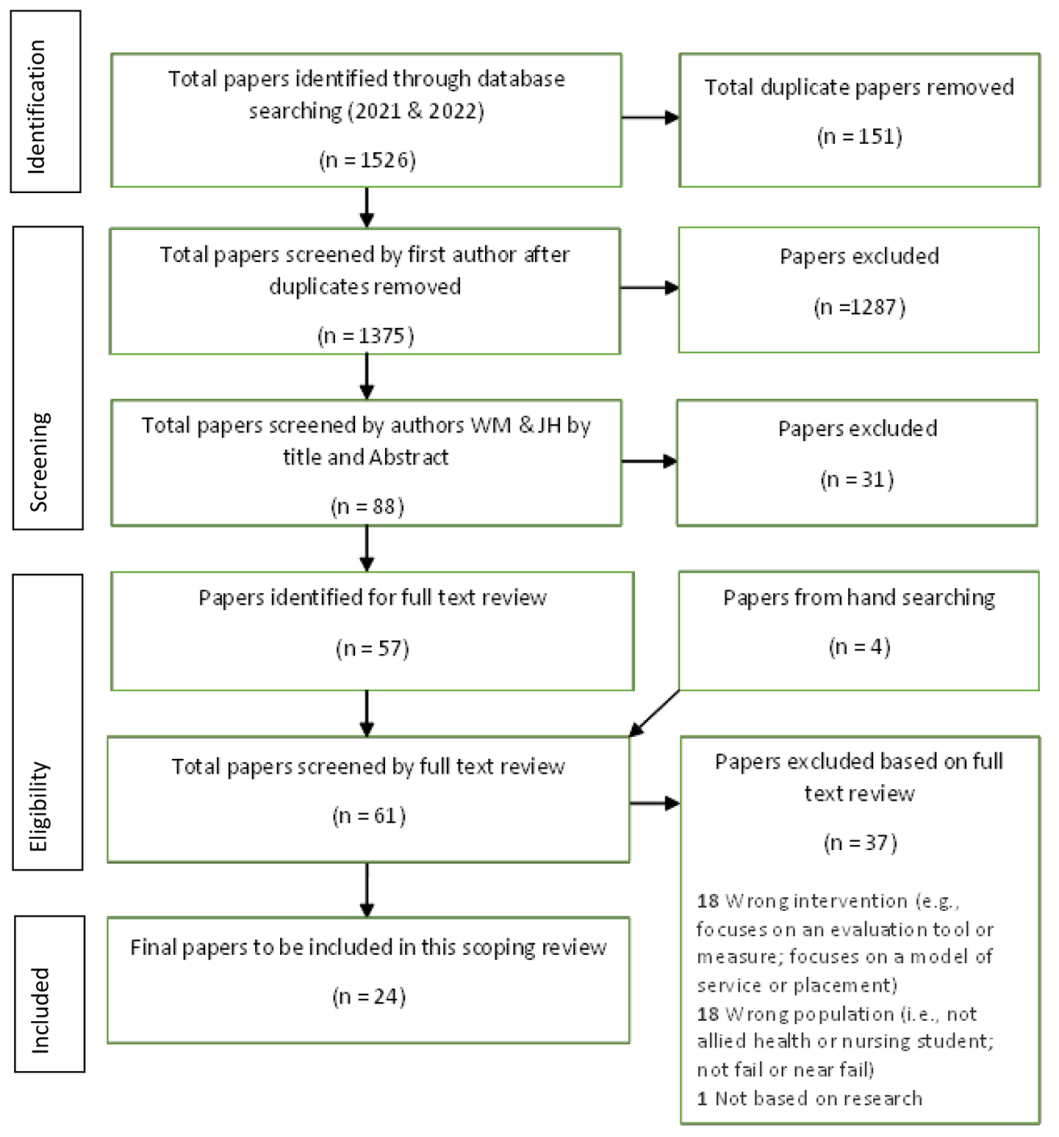
PRISMA Flowchart: Overall paper selection (incl. both June 2021 and July 2022 searches)

The PRISMA flowchart (Fig. 1) summaries the overall search results.

The initial search in June 2021 resulted in 1359 papers. The first author removed all duplicates (n = 151) and screened the title and abstracts for suitability for inclusion, resulting in 84 potential papers. Titles and abstracts of these papers were independently reviewed by two authors (WM and JH) based on the agreed selection criteria. Where there was disagreement, these papers progressed to the next stage of full-text review. Of the 84 papers, 53 papers underwent independent full text reviews by WM and JH. Any remaining disagreements were resolved by all three authors (WM, JH & JC) completing an independent full-text review, followed by discussion until a consensus was reached. Four additional papers were identified by hand searching relevant non-indexed journals and reviewing citations from included studies. In order to scan for grey literature, the first author (WM) applied similar search terms in Google Scholar and reviewed professional practice websites for potential relevant documents. No additional records were identified. The search resulted in 21 confirmed papers in July 2021. The repeated search in July 2022 resulted in 167 additional papers that were screened (title and abstract) by the first author with four of these papers progressing to a full text review by two authors (WM and JH). Three of these papers were confirmed to meet the eligibility criteria, resulting in a total of 24 final papers included.

The final 24 papers were examined by the first author (WM) who extracted information, in the form of direct quotes or paraphrased content, that related specifically to the student experience of failing a professional practice experience. To confirm that the information extracted was accurate and relevant to the research question, the other two authors independently reviewed five papers and compared this with the extracted information.

The first author then updated the extracted information from the remaining papers and sorted all statements and quotes into initial categories. Over a series of meetings, the research team reviewed, rearranged content and revised category names, then collaboratively combined these categories into broader themes. At each stage discussions continued until consensus was reached. When the themes were finalised, the number of papers that had relevant statements and quotes within each theme was counted to determine how frequent each theme was represented. As this was a scoping review, no critical appraisal of the papers was required (Peters et al., 2020).

## Results

### Nature and type of research

Tables 1 and 2 provide a summary of included papers. Of the 24 included papers, half represented social work (n = 12). Other disciplines included occupational therapy (n = 4), nursing (n = 4), physiotherapy (n = 1) and speech and language pathology (n = 1). Two papers included multiple allied health disciplines including various combinations of medicine, nursing, occupational therapy, physiotherapy and speech and language pathology.

**Table 1:** Publications included in Scoping Review

Author(s)	Year	Title	Publication	Discipline	Country
Brandon, J., & Davies, M.	1979	The limits of competence in social work: The assessment of marginal students in social work education	British Journal of Social Work	Social Work	UK (England)
Drake, V., & Irurita, V.	1997	Clarifying ambiguity in problem fieldwork placements: Picking up and dealing with problem signals	Australian Occupational Therapy Journal	Nursing Occupational Therapy Physiotherapy	Australia
Gutman, S. A., McCreedy, P., & Heisler, P.	1998	Student level II fieldwork failure: Strategies for intervention	American Journal of Occupational Therapy	Occupational Therapy	USA
Burgess, R., Campbell, V., Phillips, R., & Skinner, K.	1998	Managing unsuccessful or uncompleted placements	Journal of Practice Teaching in Social Work and Health	Social Work	UK (Scotland)
Burgess, R., Phillips, R., & Skinner, K.	1998	Practice placements that go wrong	Journal of Practice Teaching	Social Work	UK (Scotland)
Koenig, K. P.	2003	Academic and clinical success in the field of occupational therapy: Predictors of entry-level professional competence	Temple University	Occupational Therapy	USA
James, K. L., & Musselman, L.	2006	Commonalities in level II fieldwork failure	Occupational Therapy in Health Care	Occupational Therapy	USA
McGregor, A.	2007	Academic success, clinical failure: Struggling practices of a failing student	Journal of Nursing Education	Nursing	Canada
Parker, J.	2010	When things go wrong! Placement disruption and termination: Power and student perspectives	British Journal of Social Work	Social Work	UK (England)
Dove, C., & Skinner, C.	2010	Early placement breakdown in social work practice placements	Journal of Practice Teaching and Learning	Social Work	UK (England)
Attrill, S., Lincoln, M., & McAllister, S.	2012	Student diversity and implications for clinical competency development amongst domestic and international speech-language pathology students	International Journal of Speech-Language Pathology	Speech & Language Pathology	Australia & New Zealand
Poletti, A., & Anka, A.	2013	‘They thought I wasn’t good enough for social work practice’: The views of students who failed their practice learning opportunities	Journal of Practice Teaching and Learning	Social Work	Not stated
Larocque, S., & Luhanga, F. L.	2013	Exploring the issue of failure to fail in a nursing program	International Journal of Nursing Education Scholarship	Nursing	Canada
Schaub, J., & Dalrymple, R.	2013	Surveillance and silence: New considerations in assessing difficult social work placements	Journal of Practice Teaching and Learning	Social Work	UK (England)
Foote, W. L.	2015	Social Work field educators’ views on student specific learning needs	Social Work Education	Social Work	Australia
Higgins, M.	2017	Conflicting and competing roles and expectations: The conundrum of failing social work students on placements	Socialni Prace	Social Work	UK (England)
Nicola-Richmond, K., Butterworth, B., & Hitch, D.	2017	What factors contribute to failure of fieldwork placement? Perspectives of supervisors and university fieldwork educators	WFOT Bulletin	Occupational Therapy	Australia
Johnston, S., Fox, A., Coyer, & Fiona, M.	2018	Factors influencing clinical performance of baccalaureate nursing majors: A retrospective audit	Journal of Nursing Education	Nursing	Australia
Davenport, R., Hewat, S., Ferguson, A., McAllister, S., & Lincoln, M.	2018	Struggle and failure on clinical placement: A critical narrative review	International Journal of Language and Communication Disorders	Allied Health (Medicine, Nursing, Occupational Therapy, Physiotherapy, Speech & Language Pathology)	International / English Language only
So, O. W., Shaw, R., O’Rourke, L., Woldegabriel, J. T., Wade, B., Quesnel, M., & Mori, B.	2019	Clinical instructors’ experiences working with and assessing students who perform below expectations in physical therapy clinical internships	Physiotherapy Canada	Physiotherapy	Canada
McGovern, M.	2021	Assessment and decision making under the spotlight: The roles of student, practice teacher, tutor and university in four failed social work placements	Journal of Practice Teaching and Learning	Social Work	Ireland
Hughes, L. J., Mitchell, M. L., & Johnston, A. N. B.	2021	Moving forward: Barriers and enablers to failure to fail – A mixed methods meta-integration	Nurse Education Today	Nursing	Australia
Roulston, A., Cleak, H., Hayes, D., McFadden, P., OConnor, E., & Shore, C.	2021	To fail or not to fail: Enhancing our understanding of reasons why social work students failed practice placements (2015–2019)	Social Work Education (ahead of print)	Social Work	Ireland
Roulston, A., Cleak, H., Nelson, R., & Hayes, D.	2022	How power dynamics and relationships interact with assessment of competence: Exploring the experiences of student social workers who failed a practice placement	The British Journal of Social Work	Social Work	Ireland

**Table 2:** Included Scoping Review Papers | Details of Participants, Data Collection, Aim and Findings

Author (Year)	Participants	Data Collection Method/s	Aim	Results | As related to the student experience of failing professional practice
**STUDENT PARTICIPANTS ONLY**				
Gutman et al., 1998	Student academic records	Review of academic records	To identify the reasons that students failed a fieldwork experience and to evaluate the effectiveness of an intervention program for students identified to be ‘at risk’ for failing their next fieldwork experience.	The reasons for failure were categorised as being communicative or behavioural with eight sub-themes identified. An intervention program was found effective at decreasing future fieldwork failure rates.
Koenig 2003	Student academic records	Quantitative data obtained from existing student records held by the university	To identify any cognitive or non-cognitive factors that may predict fieldwork outcomes.	Students with a lower GPA were statistically significantly more likely to fail fieldwork I. Students with a lower fieldwork I score were more likely to need intervention and support to pass their fieldwork II. Students were more likely to fail fieldwork II if they had either a lower fieldwork I score, a lower GPA or English as a second language. Students with English as second language received the highest rate of intervention and support. Student’s age, gender or ethnicity was not statistically significant for predicting pass/fail or needing placement intervention and support.
McGregor 2007	Student (single case design)	Individual interview Field notes	To understand one student’s experience with failing clinical placement.	The student perceived there was a risk of being “different” and “not fitting in”. The student felt pressured to conform to the teacher’s way and to “keep quiet”. The student’s fear of further errors compromised their ability to perform competently. Student reported their relationship with their clinical educator was disconnected. The student became frustrated and resentful towards their clinical educator, felt powerless and ultimately gave up.
Parker 2010	Students	Individual Interviews	To examine student perspectives of placement breakdown and their experiences of the process.	Students felt the educator and the university have the power. Students felt powerless, lacking control and were fearful of reprisal. Students considered the process was unfair, biased against the student and impenetrable. Students desired to be involved in every step of the placement, to have access to formal and informal supports, and to be able to access impartial representation.
Poletti & Anka 2013	Students	Individual Interviews	To explore the reasons for placement failure from the student’s perspective.	Reasons for placement failure included the high expectations from educators, an unsuitable or unsupportive working environment, lack of support from the educator and/or university, and a lack of clarity regarding the assessment criteria. Students expressed a strong emotional response to failing. Students recommend having clearer assessment criteria, more information relating to what the consequences of failure would be and what supports are available if they were failing.
Johnston et al., 2018	Student academic records	Review of academic records	To identify factors that influence a student’s placement performance.	Students with a pre-entry non-health discipline, a lower GPA or were an international student were more likely to perform below standard on placement.
Roulston et al., 2022	Students	Individual Interviews	To understand the reasons for and the experiences of student’s who failed placement.	Students reported the following factors had a negative impacted on their placement experience: having a poor, strained, or unsupportive relationship with their educator, when the educator used or misused their position of power, and when the assessment and decision-making processes and outcomes were unclear and inconsistent. Students also reported their personal circumstances, health or disability impacted their performance on placement.
**STUDENTS PARTICIPANTS AND/OR UNIVERSITY STAFF AND/OR EDUCATORS (Multiple Participant Groups)**				
Burgess, Campbell, et al., 1998	Students University staff Educators	Individual Interviews	To analyse the perception, the experiences, and the implications of failing placement for students, university staff and educators. To identify the reasons why placements were unsuccessful or uncompleted.	NOTE: This paper reports on initial and interim results only. Full research data is reported in Burgess, Phillips, et al. (1998). The failing experience had an emotional, psychological, and financial impact for the student. Students felt powerless, not listened to, and unable to represent themselves in the decision-making process. Reasons for unsuccessful or incomplete placements included the student’s personal circumstances, a ‘personality clash’ between the student and educator, inadequate learning opportunities or support provided by the placement, the educator’s lack of experience, the student’s academic or writing ability, and the placement location requiring the student to travel a great distance.
Burgess, Phillips, et al., 1998	Students University staff Educators	Individual Interviews	To analyse the perception, the experiences, and the implications of failing placement for students, university staff and educators. To identify the reasons why placements were unsuccessful or uncompleted.	Students describe feeling powerless, excluded, misunderstood, not listened to, and were unaware of the formal processes and options available to them. Reasons for unsuccessful or incomplete placements included the student’s personal circumstances, a poor student-educator relationship, unsuitable placement allocation, and inadequate learning opportunities or support provided by the placement.
Dove & Skinner 2010	Student academic records University staff	Review of records from practice assessment panel Individual interviews (with university staff)	To identify the reasons for placement failure.	Reasons for failure were due to a complex mix of interrelated factors, including the student’s health and personal circumstances, the student’s immaturity or lack of professionalism, a lack of availability and support from the educator, and a poor student-supervisor relationship.
Higgins 2017	Students University staff Educators	Individual interviews Focus Groups	To determine if students are failing placement due to the expectations of stakeholders of students on placement.	Organisational and professional expectations of each participant group can be contradictory and inconsistent which impacted the student’s ability to meet expectations for placement.
McGovern 2021	Student academic records	Review of placement documentation and evaluations	To investigate the role, actions, and experiences of key stakeholders when a student fails placement.	Failing can be a traumatic and emotional experience for everyone involved - the student, the educator, and the university staff. Findings revealed that the student’s health and disability can impact performance, any performance concerns need to be explored and addressed in a timely manner and feedback needs to be clear and consistent. Found that university staff should provide mentorship and support for students and that students can pass subsequent placements.
**UNIVERSITY STAFF AND/OR EDUCATOR PARTICIPANTS – EXCLUDING STUDENTS**				
Brandon & Davies 1979	University staff	Individual Interviews Review of reports and attendance at examination board	To explore the experiences of educators and university staff when determining pass/fail for students who are at risk of failing.	Eight unique categories were identified as to why students failed representing communication, professionalism, knowledge, and interactions with clients and colleagues. The assessment was considered complex due to the student’s resources, skills and circumstances that may impact the student’s performance. They recommend clearer assessment processes and performance expectations.
Drake & Irurita 1997	Educators	Individual Interviews	To explore the educator’s experience of working with problem students.	According to educators, problem students were often characterized by those who demonstrated poor communication skills, unprofessional behaviours, unsafe practices, had difficulties with integrating knowledge and problem-solving, lacked motivation or engagement, or denied there were any performance problems, or had personal circumstanced impacting their ability to perform on placement. Educators acknowledged the supervisory relationship is intense and time limited. The educators expressed uncertainty or lack of clarity regarding their supervisory role and the process of working with problem students. Educators are challenged by wanting to be aware of a student’s past performance issues whilst trying to avoid any potential bias or breach of confidentiality.
James & Musselman 2006	Educators	Questionnaire Individual interviews	To identify commonalities in failing placement relating to student characteristics, the supervisory structure, and how it was addressed.	Some common student characteristics reported in relation to failing placement include inadequate academic preparation, poor clinical skills, safety concerns, judgement errors, poor clinical reasoning, difficulty responding to feedback, difficulty grasping the big picture, and poor organizational skills. Supervisors provided both written and verbal feedback, were present to provide guidance and assistance and most commonly the student had more than one supervisor involved in their placement and evaluation. Students were informed first of their performance issues, with the university program typically being informed the following week. Most times the university became involved with the student and educator but not always.
Attrill et al., 2012	University staff	Survey	To identify the performance levels and perceptions of placement performance for international Speech and Language Pathology students.	Domestic students experienced statistically significant lower rates of placement failure than international students. Undergraduate domestic students were significantly less likely to require additional placement support. Undergraduate domestic students required significantly fewer supplementary (extended or repeated) placements. Students from a non-English speaking background or non-western cultural background may experience greater difficulties on placement. International students were more likely to be rated as having difficulties in communication, as well as in the professional, group, and community education competencies on the COMPASS student evaluation tool.
Larocque & Luhanga 2013	University staff Educators	Individual Interviews Focus group	To explore the issue of ‘failure to fail’ in a nursing program.	Failing has consequences for all stakeholders i.e. the student, the agency and the university. Failing a student is a difficult process and students and educators require academic and emotional supports.
Schaub & Dalrymple 2013	Educators	Individual Interviews	To research educator’s experiences and views of students who are challenging or failing on placement.	Students demonstrated several challenges, such as poor communication skills, inadequate engagement with the team and service users, limited insight, and insufficient capacity for reflection. Additionally, they were unable to demonstrate their professional identity and values, specific to the discipline.
Foote 2015	University staff Educators	Focus groups	To identify common learning issues experienced by students who are having difficulties on placement.	Difficulties during placement arose due to various reasons, such as the student’s health or disability, inability to transition into a professional role, and an ineffective use of supervision. Suggested strategies to address these issues included improving the student-placement match, increasing disclosure of factors relating to a student’s health and well-being and improving student preparations for entering a professional context for placement.
Nicola-Richmond et al., 2017	University staff Educators	Individual Interviews Survey	To explore participant’s perspectives of the contributing factors for students failing placement.	Failing a student is a difficult and time-consuming experience for the educator. Reasons for students failing placement included poor communication and reflection skills, nondisclosure of health issues and an inability to accept feedback. Findings highlighted the importance of having a strong student-educator relationship with clear and regular communication, supervision, and feedback. Recommend that issues should be identified and addressed as early as possible, that disclosure of considerations relating to the student’s health and well-being is desirable and additional support is needed for students with English as their second language.
Davenport et al., 2018	n/a	Critical narrative review	To review the research regarding failing and struggling health professional students undertaking clinical placements with a focus on Speech and Language Pathology students.	Most research found in this review represented medicine and nursing with only a few publications representing allied health. The voice of the struggling student was largely absent in the literature. Research in this area focused on aspects such as the identification of at-risk students, support and remediation strategies, the humanistic nature of learning, the concept of failure to fail or the impact of policy and processes. Further research is needed that combine both the predictive or risk factors along with remediation strategies and not just exploring these factors independently.
So et al., 2019	Educators	Individual Interviews	To explore the experiences of educator’s and their decision-making process relating to supervising students who are performing below expectations on placement.	The educator would appreciate disclosure about a student’s performance or other learning considerations before the placement commenced. Early and honest communication about performance concerns is of value. A student would fail if there were repeated incidents, not due to a single incident or event. The student’s ability to respond to and implement feedback had the greatest impact on the educator’s assessment and recommendations. Educators wished university had followed up and advised them of the assessment outcome / final decision.
Hughes et al., 2021	University staff Educators	Individual Interviews Survey	To explore and further understand the enablers and barriers for educators when determining a pass or fail outcome for students on placement.	Enablers for educators to fail a student include having access to their own (educator) supports, being in an organisation that supports failing a student and the program is flexible and able to provide the student with alternative or additional learning opportunities. Educators are less likely to fail a student if the educator tends to rate a student’s performance higher than is deserved or they may give the student the benefit of the doubt. Other barriers to failing a student include if the educator is concerned about or have had experienced negative or inappropriate student responses, if the organisational process for failing a student is considered cumbersome and burdensome and if the workload and time associated with failing a student is too high and is difficult to manage for the educator.
Roulston et al., 2021	Student academic records Educators	Review of placement documents and practice assessment panels	To identify the incidence of, and the reasons why students fail professional practice (as reported by educators).	The fail rate for social work students in Ireland was 3%. Four categories for failure were identified including the student’s skills, knowledge, their values or personal factors. The four highest specific reasons that were identified was that the student demonstrated either a lack of understanding of their professional role, had poor time management or poor writing skills or being unable to follow guidance.

The UK was represented in seven studies and Australia in five. Ireland, Canada, and the USA were the focus of three papers each and the origin of one other paper was not stated (n = 1). One other paper combined results from Australian and New Zealand participants. One paper was a review of which included international (English language only) studies  (Davenport et al., 2018). Most papers were published after 2010 (n = 16; 67%), with four published in 2021.

Most of the papers used a qualitative design and typically involved individual semi-structured interviews (n = 17) or focus groups (n = 2); or reviewed the student’s academic records or placement-related documentation (n = 7). Two studies were individual student case studies (McGovern, 2021; McGregor, 2007) and only one was a critical narrative review (Davenport et al., 2018). Of the studies that conducted interviews and/or focus groups, smaller sample sizes of 7 to 25 participants were common (n = 13; 54.2%) (Attrill et al., 2012; Dove & Skinner, 2010; Drake & Irurita, 1997; Foote, 2015; Hughes et al., 2021; James & Musselman, 2006; Larocque & Luhanga, 2013; Nicola-Richmond et al., 2017; Parker, 2010; Poletti & Anka, 2013; Roulston et al., 2022; Schaub & Dalrymple, 2013; So et al., 2019).

As highlighted in Table 2, only seven (29.2%) of the 24 papers had students or student data as their only participant group (Gutman et al., 1998; Johnston et al., 2018; Koenig, 2003; McGregor, 2007; Parker, 2010; Poletti & Anka, 2013; Roulston et al., 2022) with an additional five papers also including educators or university staff (Table 2: Multiple participant groups) (Burgess, Campbell, et al., 1998; Burgess, Phillips, et al., 1998; Dove & Skinner 2010; Higgins, 2017; McGovern, 2021). Of these 12 papers that included student participants, social work was the most represented discipline (n = 8). Importantly, half of the papers (n = 12; 50%) did not include any students as participants (Table 2: University Staff and/or Educator Participants – excluding students).

### Themes identified

Six themes emerged in relation to student experiences of failing professional practice: (1) a multifaceted matrix of reasons; (2) the look and feel of failing; (3) missing the mark – the influence of supervision, supports, services and strategies; (4) the whos and the hows – the importance of communication, relationships and organisational culture; (5) powers at play – the impact of infrastructure, policies and procedures; and (6) failing has consequences.

### Theme 1: a multifaceted matrix of reasons

A majority of the papers (n = 17; 70.8%) investigated the reasons why students had failed, however only seven of these clearly articulated that these were from the perspectives of the students (Burgess, Phillips, et al., 1998; Dove & Skinner 2010; Gutman et al., 1998; Higgins, 2017; McGovern, 2021; Poletti & Anka, 2013; Roulston et al., 2022). Within these 17 studies, only in two papers did students report that their own personal circumstances such as their health or family and caring commitments had impacted their ability to cope and perform well on placement (Dove & Skinner, 2010; Roulston et al., 2022). These factors were however more commonly cited by the educators or university staff (n = 7) who reported that the student’s performance on placement was regularly impacted by their personal responsibilities (i.e. family, carer or employment demands), or the student’s general health, mental health or an identified disability (Brandon & Davies, 1979; Burgess, Campbell, et al., 1998; Burgess, Phillips, et al., 1998; Foote 2015; James & Musselman, 2006; Nicola-Richmond et al., 2017; Roulston et al., 2021).

In contrast, students believed that their performance issues were due to a lack of support from their educator, being unable to meet the high or unrealistic expectations set by their educator, or their own uncertainty about the assessment criteria (Dove & Skinner, 2010; Higgins, 2017; McGovern, 2021; Poletti & Anka, 2013). Some students believed that their educator was unprepared or lacked supervision experience (Gutman et al., 1998; Parker, 2010). Students also commented on the overall impact of the working environment on the quality of their placement, but no further insights were provided (Burgess, Campbell, et al., 1998; Parker, 2010; Poletti & Anka, 2013).

Thirteen papers explored performance-based reasons for students failing (54.2%). Educators or university staff reported that performance-based reasons for failure included students having difficulties demonstrating practical skills, communicating with clients or colleagues, or being able to problem solve and professionally reason (Attrill et al., 2012; Brandon & Davies, 1979; Burgess, Phillips, et al., 1998; Drake & Irurita 1997; Gutman et al., 1998; James & Musselman, 2006; Nicola-Richmond et al., 2017; Roulston et al., 2021; Schaub & Dalrymple, 2013; So et al., 2019). They also reported that students were not able to act on feedback, engaging in supervision in a limited way, and demonstrating a general lack of insight into their performance deficits (Burgess, Phillips, et al., 1998; Drake & Irurita 1997; Foote, 2015; Gutman et al., 1998; James & Musselman, 2006; McGovern, 2021; Nicola-Richmond et al., 2017; So et al., 2019). Only on one occasion did a student state that their failure in communication skills was due to English being their second language (Poletti & Anka, 2013). Educators also identified that students who have English as their second language demonstrated difficulties in their written and verbal communications during placement that required a failure evaluation (Attrill et al., 2012; Foote, 2015; Nicola-Richmond et al., 2017). Students did not report on performance-based reasons for their failure.

### Theme 2: the look and feel of failing

The second most common theme described in 16 of the papers (66.6%) encapsulated the student’s behavioural and emotional responses to failing. However, only five papers clearly identified that this information was obtained from the students themselves (Burgess, Phillips, et al., 1998; McGregor 2007; Parker, 2010; Poletti & Anka, 2013; Roulston et al., 2022).

A range of emotions when faced with failing or potential failure were identified. Students expressed that they felt embarrassed and ashamed (Roulston et al., 2022), disappointed (Poletti & Anka, 2013; Roulston et al., 2022), frustrated, angry (McGovern, 2021; McGregor, 2007) sad, tearful, anxious (Dove & Skinner, 2010; Poletti & Anka, 2013) and emotionally drained (Poletti & Anka, 2013). One student described that the experience had a negative impact on their physical health, reporting feeling physically sick and not being able to eat both during and after the fail experience.

*I felt like dying. I felt horrible. It was Christmas and it was the worst Christmas I’ve ever had. I was not eating and I had a medical issue as well so that complicated things for me…. I felt horrible …’ I’ve got a little boy who knows I go to college; he knows I work hard... now I had to tell him ‘You know what? Mummy’s not going to be a social worker* (Poletti & Anka, 2013, p. 26).

One student describes experiencing *‘a complete breakdown’* after their fail experience and a second student spoke of becoming dependent on alcohol *“the local off-licence did very well out of me”* (Burgess, Phillips, et al., 1998, p. 53).

Educators and/or university staff also described observing a similar range of emotional responses from students including students being tearful, frustrated, angry, shocked, confused, sad and/or depressed (Burgess, Phillips, et al., 1998; Dove & Skinner 2010; James & Musselman, 2006; Larocque & Luhanga, 2013; McGovern, 2021). They also experienced students responding with denial, blame, aggression, retaliation, intimidation or defensiveness (Dove & Skinner, 2010; James & Musselman, 2006; Schaub & Dalrymple, 2013).

*[The student] became sort of aggressive – you know, that sort of silent aggression? Intimidation like, you know, ‘What are you doing failing me?’* (Schaub & Dalrymple, 2013, p. 91).

Students expressed that failing made them feel like giving up (McGregor, 2007) and started questioning their overall career choice and skills.

*“I felt very useless and I felt that I was no good for the job because all the other students were successful and I wasn’t. I started asking myself a lot of questions”* (Poletti & Anka, 2013, p. 27)

Students described that failure had led to a reduced confidence and negatively impacted their self-esteem (Parker, 2010; Roulston et al., 2022). They further reflected that this lack of confidence then contributed to them not being able to demonstrate any initiative to improve their performance during placement (Parker, 2010).

After students became aware they were failing, educators reported similar observations of the student’s behaviour. Educators believed that as the student’s self-esteem and confidence deteriorated, this resulted in students not being able to actively engage in any learning activities or identify and implement the required changes (Burgess, Phillips, et al., 1998; James & Musselman 2006; Larocque & Luhanga, 2013; So et al., 2019). They sensed that students felt alienated, excluded, or unwelcome, and they observed students actively withdrawing or disengaging from the team and others (Burgess, Phillips, et al., 1998; Dove & Skinner 2010; Higgins, 2017). They believed that students became unmotivated to change (So et al., 2019) and some students continued to demonstrate a lack of insight into their performance issues (Drake & Irurita, 1997; James & Musselman, 2006; McGovern, 2021; Nicola-Richmond et al., 2017; So et al., 2019).

Students, however explained the reasons for these behaviours in different ways, reporting feeling powerless (Burgess, Campbell, et al., 1998; Burgess, Phillips, et al., 1998; Dove & Skinner 2010; Higgins, 2017; McGregor, 2007; Parker, 2010) and oppressed by the system (Burgess, Phillips, et al., 1998). One student expanded further, stating that they felt misunderstood, disregarded, not trusted or respected by their educator and felt pressured by always being ‘assessed’ or ‘watched’ (McGregor, 2007). This student added that once they knew they were failing, they developed a heightened paranoia about making future errors, which led to further deterioration in their performance.

*“I don’t think I ever lost that paranoia of “Oh my god! What if I do something else wrong?... It was such a huge stumbling block for me to be hit with this confidence problem... If I made one more error, any error, she will send me off the floor... Now I am so scared... that was interpreted as [my] being lazy and disinterested...”* (McGregor, 2007, p. 508).

During the placement, students described feeling fearful of further negative consequences if they were to raise any concerns regarding the quality of their placement or to challenge any feedback or evaluations. This created a feeling of vulnerability as they perceived that any dissent from them could further compromise their evaluation (Burgess, Phillips, et al., 1998; Higgins 2017; Parker, 2010).

After the placement was finalised, in terms of behaviours, some students were reported by educators and/or university staff to have responded by submitting negative reviews of teaching staff, posting inappropriate comments on social media, making accusations of bullying and harassment, formally appealing the decision or threatening legal action (Dove & Skinner, 2010; Hughes et al., 2021; James & Musselman, 2006; Schaub & Dalrymple, 2013).

### Theme 3: missing the mark: the influence of supervision, supports, services and strategies

This theme describes how the supervision, supports, services or strategies provided during placement influenced a student’s placement experience, and how this may have impacted their performance outcomes. When reflecting upon reasons contributing to their performance difficulties, students spoke about the lack of support from and the availability of their educator (Dove & Skinner, 2010), not receiving regular supervision (Dove & Skinner, 2010), being unfairly treated (Dove & Skinner, 2010; Gutman et al., 1998) not listened to (Burgess, Phillips, et al., 1998), and receiving inconsistent feedback or being unclear of the expectations (Dove & Skinner, 2010; Gutman et al., 1998; Roulston et al., 2022). Only one study reported that students believed that a lack of structure and the limited or poor learning opportunities provided during placement contributed to their failing performance (Parker, 2010). Only in one case did a student identify that their educator provided the positive support they required (Parker, 2010). Only in four studies did university staff or educators acknowledge that the failing outcome may have been due to the educator lacking supervision skills (Burgess, Phillips, et al., 1998) or being unsupportive (Burgess, Phillips, et al., 1998; Dove & Skinner 2010; Higgins, 2017).

A key role for university staff is to provide support to underperforming students as well as act as a liaison and support for educators (Stutz-Tanenbaum et al., 2015). In two studies, students reported feeling unsupported by the overall structure of the university system (Burgess, Phillips, et al., 1998; Poletti & Anka 2013). However, they still considered the university staff to be a positive source of support if that staff member was knowledgeable about the university program and the placement requirements, and behaved in a manner that the student considered to be proactive, accessible and demonstrated an honest, fair and transparent use of the staff member’s authority (Parker, 2010; Poletti & Anka, 2013).

Early identification of performance issues and communicating this to the student was considered highly important by all stakeholders, to allow enough time for the student to address deficits and implement strategies to improve their performance (Burgess, Phillips, et al., 1998; Dove & Skinner 2010; Drake & Irurita, 1997; Foote, 2015; James & Musselman, 2006; Nicola-Richmond et al., 2017). However, students in some studies reported that performance issues were not highlighted to them until too late in their placement, that they didn’t receive adequate warning (Burgess, Phillips, et al., 1998) and they were not given notice about the possible termination of their placement (Dove & Skinner, 2010).

When performance issues were identified, common strategies facilitated by university staff and/or educators included issuing formal written notices to the student regarding their poor performance in the form of ‘constructive notes’, creating formal written action plans in the form of learning contracts or remediation plans, or providing additional learning opportunities such as role-playing clinical scenarios (Davenport et al., 2018; Gutman et al., 1998; Johnston et al., 2018; Koenig, 2003; So et al., 2019). University staff and educators indicated that these strategies were considered beneficial as they clarified to the student the specific expectations and performance outcomes to be met within a designated timeframe (Davenport et al., 2018; Gutman et al., 1998; Koenig, 2003). No papers in this review explored the student’s perceptions of these strategies and whether they considered them desirable, timely or effective.

In relation to other supports sought, accessed, or desired, one student described their need for further formal and informal supports including a desire for an independent forum or person to be involved (Parker, 2010). Another student expressed a need for additional placement support in relation to English being their second language, but reported that this support was not offered (Poletti & Anka, 2013).

Gutman et al. (1998) was the only study that used pre-placement interventions including seminars, mentoring and community volunteering for those students that university staff identified to be ‘at risk’ of failing their next placement. Of the ten students who attended the program, seven were successful in their next placement. This study considered the program to be effective at reducing the rate of failing future placements.

### Theme 4: the whos and the hows: the importance of communication, relationships, and organisational culture

Educators demonstrate various communication styles and how they develop and maintain relationships with other stakeholders impacted on the student’s experiences. Students in the study by Dove and Skinner (2010) described their educator’s communication style as negative, critical and lacking encouragement or clarity. Students also described in several other studies that having a negative or poor relationship with their educator was detrimental to their ability to succeed, and a barrier to feeling safe enough to honestly disclose any difficulties they were having on placement and the reasons for these (Burgess, Phillips, et al., 1998; Dove & Skinner 2010; Roulston et al., 2022).

University staff and educators similarly commented on the student-educator relationship, also indicating that poor relationships with educators may have made students feel disconnected or unsafe and consequently reluctant to disclose or discuss any perceived performance issues (Foote, 2015; Schaub & Dalrymple, 2013; So et al., 2019). In some studies, it was noted by students and educators alike that the relationship further deteriorated as issues were raised, and consequently student engagement and performance worsened for the remainder of the placement (Burgess, Phillips, et al., 1998; Dove & Skinner 2010; Schaub & Dalrymple, 2013).

The nature of the placement environment at times also impacted the student’s performance. One study by Parker (2010) identified that students had identified concerns about the agency or management’s overall commitment to practice education and how learning opportunities were allocated. A similar concept was identified in the study by Higgins (2017), where students describe an organisational culture in which they were not treated seriously as they were “not practitioners”; experiencing an “us” [students] verses “them” [educators] culture where students were talked about and not with.

### Theme 5: powers at play: the impact of infrastructure, policies and procedures

The way in which placements are coordinated and supported is primarily governed by the university program in consultation with agency requirements. This includes how placements are sourced, allocated, managed and monitored, how an underperforming or failing student is managed both during and after the placement and how a failing result is confirmed (Stutz-Tanenbaum et al., 2015).

Students commented that they were aware of limited placement offers due to the growing demand for student placements. Students from one study explained that this knowledge made them feel additional pressure to not withdraw or decline a placement allocation, nor question the quality of their placement even when they were unhappy with their allocation, or when they experienced difficulties on placement. This reinforced their sense of powerlessness and disempowerment (Burgess, Campbell, et al., 1998; Burgess, Phillips, et al., 1998; Parker 2010). Students in the Parker (2010) study further articulated that they believed their individual needs were not considered when being allocated to a placement, resulting in a poorer placement allocation and monitoring experience. The suitability of the student-placement allocation was only mentioned by educators in two papers as a potential contributing factor to a student’s failing performance (Dove & Skinner, 2010; Foote, 2015).

In relation to student performance evaluations, some students reported that they were unaware of the expectations and assessment criteria for placement and how their performance was being assessed (Parker, 2010; Poletti & Anka, 2013; Roulston et al., 2022). Students were unclear or confused about the supports available and the associated process when performance concerns were identified or a fail grade was finalised. This included the presence and purpose of any appeal systems and how to action these (Burgess, Phillips, et al., 1998; Roulston et al., 2022).

When performance issues were identified, some placements implemented an additional assessor for a ‘2nd opinion’, a process with which students reported contrasting experiences. At times, this was considered by students as helpful, albeit reaffirming of their performance issues (Burgess, Phillips, et al., 1998; Parker 2010). There were mixed experiences from students in the study by Burgess, Phillips, et al. (1998) where some students identified that an additional assessor would have been welcomed but was not offered; another student reported that they did have a second assessor which they considered helpful; whilst a third student stated that this process caused them increased stress and anxiety, leading to a further deterioration of their performance. Students in the Parker (2010) study reported that having a second assessor was not at all helpful, questioning the integrity of this process and expressing that they felt the university and the educators were colluding against them. Students also considered that this process was, at times, implemented too late (Parker, 2010; Roulston et al., 2022).

In social work programs in England and Scotland, when a student ‘failed’ their placement as determined by their educator, a formal post placement review panel was conducted by university staff to make a final determination of the outcome (Burgess, Phillips, et al., 1998; Parker 2010). Students spoke negatively about this panel, stating that they felt excluded and unable to represent themselves, and that the decision-making process was inflexible, unsupportive, unilateral and an “exercise in power” by the university (Burgess, Campbell, et al., 1998; Burgess, Phillips, et al., 1998; Parker 2010, p. 992). Consequently, they felt disempowered, humiliated, and suspicious, feeling that they were not able to adequately represent themselves in this final decision-making process (Burgess, Campbell, et al., 1998; Parker, 2010).

### Theme 6: failing has consequences

Only three papers articulated the consequences of failing for the students themselves. Burgess, Campbell, et al. (1998) reported that the failure experience had a considerable emotional, psychological, and financial toll on students with the experience described as ‘traumatic’. This experience was stated to have impacted the student’s overall lives, careers and the people who were close to them. It is unclear as to whether this was information obtained from the students themselves or the other participant groups in that study. The other study by Burgess, Phillips, et al. (1998) reported that one student had lost employment as a direct consequence of failing and not being able to obtain their qualification in time. Similarly, the Larocque and Luhanga (2013) paper reported that educators and university staff believed that while these students still wanted to complete the program and become qualified, the fail had caused them to lose time and money.

## Discussion

We sought to better understand allied health and nursing student’s experiences of failing or near failing a professional practice experience. The findings of this scoping review suggest three key characteristics of the research to date: (a) the student voice is still largely missing; (b) the student perspective is distinctly different to that of other stakeholders; and (c) the interventions used appear not to be student-informed or student-led.

The lack of student representation on this topic has been acknowledged across the years (Burgess, Campbell, et al., 1998; Burgess, Phillips, et al., 1998; Lew et al., 2007; Parker, 2010), with the most recent critical narrative by Davenport et al. (2018) highlighting the need for further studies with allied health students as participants. Our review revealed that research including the student voice is dominated by social work and predominantly from the UK or Ireland. We identified only two papers published since 2018 who had student participants, indicating that contemporary literature with student participants is lacking (McGovern, 2021; Roulston et al., 2022). Our findings confirm that the student’s voice continues to be largely under-represented for most other allied health disciplines.

This gap may be due to several factors. For students to engage as research participants they need to trust the researcher and feel that their contribution will be valued and worthwhile (Manohar et al., 2019). Our findings reveal that students at times felt powerless and untrusting of the university in relation to how their failure was managed. Consequently, students may be reluctant to engage in any university-facilitated research in this field.

Failing placement has also been identified as an emotional and traumatic experience (Bearman, 2020; Burgess, Campbell, et al., 1998), often associated with shame or embarrassment (Roulston et al., 2022). Consequently, accessing student participants that are actively undergoing or have recently undergone a failure experience in a timely and sensitive manner may be difficult for researchers to achieve. In addition, the low numbers of students that fail placement each year (American Occupational Therapy Association, 2018; Foote, 2015; Johnston et al., 2018) may limit the number of potential student participants.

Another important finding from this scoping review is that when student perspectives on failing placement were gathered, these were distinctly different to that of other stakeholders. Firstly, the underlying contributing factors for failing the placement as identified by the student often contrasted to those factors reported by educators or university staff. Students often identified factors that related to the educator’s skills or the educators having unclear or high-performance expectations of the student (Gutman et al., 1998; Parker, 2010; Poletti & Anka, 2013). Educators and university staff rarely identified these factors, instead focusing on the impact that a student’s health, disability or personal circumstances had on the student’s ability to actively engage and perform in the placement context (Brandon & Davies, 1979; Burgess, Campbell, et al., 1998; Burgess, Phillips, et al., 1998; Foote 2015; James & Musselman, 2006; Nicola-Richmond et al., 2017; Roulston et al., 2021).

Whilst there were some commonalities in how all stakeholders believed students felt and behaved throughout the failing experience, students provided a unique perspective to explain these responses. The student’s emotional response, their feelings of disempowerment and a poor student-educator relationship all appear to have influenced how motivated and engaged the student was to proactively seek support or to contribute to the development and implementation of any possible remediation strategies.(Roulston et al., 2022)(Burgess, Campbell, et al., 1998; Dove & Skinner, 2010; Roulston et al., 2022).

Social determination theory (Ryan & Deci, 2017) may help to explain these responses. This theory states that, to be motivated, a person needs to feel a sense of competence, autonomy and relatedness towards the goal. When a context does not adequately support these elements, or does so in a negative way, this can lead to people feeling a-motivated, and becoming aggressive, defensive, antisocial or ineffective (Ryan & Deci, 2017). These behaviours were all observed from students in the included studies in relation to a failing performance.

In self-determination theory, for a person to be motivated to make any behavioural changes required to achieve a goal, this goal should be intrinsically desirable for the person, and not driven by external rewards, evaluation or punishment (Deci & Ryan, 2000). We could assume that students have the goal of developing and demonstrating the required skills to pass their placement. However, as they are being formally evaluated by an external party (i.e., the educator), when performance issues are raised, a student’s sense of autonomy or control over the outcome is likely to become compromised. Therefore, to counteract this potential loss of autonomy, it is important that the student remains central to any decision-making process regarding how to address the performance deficits so that they have the best chance of staying motivated towards achieving their goal of passing.

The role of the university and its professional practice staff in managing and supporting students who are failing or struggling on placement is recognised (Power & Albaradura, 2018; Ruth & Marguerita, 2014; Stutz-Tanenbaum et al., 2015). Yet, in the studies included in this review, students often reported being unclear and confused about the assessment criteria, the relevant policies and procedures, and what they should or could do in relation to failing placement, including the appeals process (Burgess, Phillips, et al., 1998; Parker 2010; Poletti & Anka, 2013; Roulston et al., 2022).

Whilst access to university policies and tools may be available, students may not appreciate the importance or relevance of this information at the time it is presented to them. It is likely that it is not until they start to experience difficulties or fail the placement that they will seek this information. University programs therefore could ensure that students remain appropriately informed throughout the placement experience so that information is targeted and relevant to each student’s circumstances at the time.

When a student was determined to be underperforming or failing during their placement, a range of actions were implemented to address these performance issues, with learning contracts being the most common tool mentioned. Implementing learning contracts has been demonstrated to improve a student’s engagement and motivation in the learning process (Swartz, 2019). Learning contracts enable students to be active and autonomous learners, facilitating a more collaborative learning experience that establishes and maintains mutual trust and respect whilst recognising the student’s individual needs and preferences in learning (Rye, 2008; Whitcombe, 2001). This is only the case however, if students are the active and leading agent in the contract’s development and implementation (Matheson, 2003; Rye, 2008; Swartz, 2019).

Unfortunately, when used to address performance issues on placement, these strategies appear to be typically initiated and implemented by the educator or the university (Bearman, 2020; Turkett, 1987). Further exploration of the student’s perceptions of these interventions is warranted to determine if these are truly student-centred and implemented in a way that the student considers effective, or if alternative strategies are desired.

After a failing experience, students may be offered the opportunity to repeat a placement. Students usually go on to successfully pass these future opportunities and progress to course completion and graduation (Gutman et al., 1998; McGovern, 2021; Roulston et al., 2022). How to prepare and support students for this next professional practice experience was rarely discussed in the literature, with only one study by Gutman et al. (1998) evaluating a pre-placement intervention program for occupational therapy students considered ‘at risk of failing’. Whilst considered effective at the time for reducing future failure on placement, it is unknown if this program continues to be offered and continues to be effective, or if other strategies or programs are now being implemented.

Students are likely to be concerned about potentially failing their next placement experience, commencing their next placement with a reduced level of self-esteem and confidence and a heightened sense of anxiety and apprehension (Ruth & Marguerita, 2014). The impact of the clinical learning environment on a student’s ability to perform effectively was highlighted in our findings, with the role of the educator and their relationship with the student a key contributing factor in the student’s performance. An educator considered by the student to be supportive and encouraging had a strong influence on the student being able to regain their confidence in the professional practice context (McGregor, 2007; Parker, 2010; Ruth & Marguerita, 2014). How university programs support students to prepare for these repeat placements needs further investigating to maximise the chance of future placement success.

This scoping review has identified some important implications for practice for educators and universities alike across several elements. Firstly, how existing systems and processes are experienced and perceived by students needs to be addressed. Current practices appear to be resulting in a sense of vulnerability and a power differential that impedes a student’s autonomy, motivation, and control in times of failing or near failing and restricts a student’s willingness to seek further support and advocacy in these times.

How one is interpreting the student’s behaviours is encouraged to be explored, as alternative reasons have been presented by students in these studies. Remediation strategies and supports will continue to be ineffective until this common understanding is established.

The importance of the student-educator and the student-university relationship has also been highlighted as a key influencer of the student experience along with the quality and timing of feedback on a student’s performance. All stakeholders need to be familiar with their role in establishing and fostering positive relationships and providing and responding to feedback, particularly in times where a performance issue has been identified and raised. All stakeholders should be aware of how their behaviour contributes to this relationship, and how they can address any issues in these relationships if they are deteriorating or get to the stage of being beyond repair. These elements can easily be included in any relevant training or resources that is currently being provided.

It is clear from our review that further research is needed to increase the student voice to better understand their experiences of failing or near failing professional practice experiences across most allied health disciplines. By better understanding the contemporary student experience of failing, we would aim to reduce the impact this has on all stakeholders by designing and delivering more effective and sustainable solutions to address the student’s needs. By fostering a sustainable practice education environment to better support students, we can ensure they continue their journey towards becoming qualified and competent allied health professionals.

## Limitations of the review

Inherent in scoping reviews, the quality of the included literature was not required to be assessed (Arksey & O’Malley, 2005). While the authors followed the necessary steps to complete a scoping review, the transferability of the findings in this paper is limited by the variability in terminology related to the topic. For example, the search term ‘clinical education’ was considered but subsequently excluded due to the generation of a significant number of irrelevant results that related to the graduate experience. While databases covering a range of allied health professions were utilized in this study, the limitations of the authors’ decision not to individually identify all professions that could be defined as allied health in the search strategy should be considered as this may have resulted in all relevant papers not being identified. Additionally, the exclusion of papers focusing solely on interventions, screening for ‘at-risk’ students, or ‘academic’ failure may have also limited the potential to find further relevant results. Finally, there is a possibility that relevant papers or information from grey literature may have been missed. Despite these limitations, this review has shed some light on the thoughts and perspectives of allied health and nursing students dealing with placement failure and provides educators and university staff with an alternative viewpoint as to why a student may appear disengaged, withdrawn, or unmotivated to change.

## Conclusion

Students respond in a variety of ways when failing or near failing a professional practice experience. The student’s voice regarding this experience is often missing, and when captured, contrasts with reports from other stakeholders. Inadequate understanding of the student experience may impact the effectiveness of any supports or solutions offered as these may not address the student’s identified needs or priorities. This gap in contemporary knowledge of the student perspective needs to be addressed so supports can be developed and implemented from a student-led or student-informed lens.

## Appendix

**Table:** Appendix: Final CINAHL Search Strategy (Literature Search performed – 26 June 2021)

S4 AND S5 AND S6	Narrow by Language: - english
S4	S1 AND S2 AND S3
S3	fail* OR at risk
S2	fieldwork OR student placement OR professional practice OR work integrated learning
S1	undergraduate students OR (MH “students, health occupations”) OR allied health students OR occupational therapy students OR physical therapy students OR speech-language pathology students OR social work students OR pre-entry
